# Expansion of primary healthcare and emergency hospital admissions among the urban poor in Rio de Janeiro Brazil: A cohort analysis

**DOI:** 10.1016/j.lana.2022.100363

**Published:** 2022-09-05

**Authors:** Thomas Hone, James Macinko, Anete Trajman, Raffaele Palladino, Claudia Medina Coeli, Valeria Saraceni, Davide Rasella, Betina Durovni, Christopher Millett

**Affiliations:** a Public Health Policy Evaluation Unit, Imperial College London, London, United Kingdom; b Departments of Health Policy and Management and Community Health Sciences, Jonathan and Karin Fielding School of Public Health, University of California Los Angeles, United States; c Federal University of Rio de Janeiro, Brazil; d Department of Public Health, University “Federico II” of Naples, Italy; e Instituto de Estudos em Saúde Coletiva, Universidade Federal do Rio de Janeiro, Rio de Janeiro, Brazil; f Secretaria Municipal de Saúde do Rio de Janeiro, Rio de Janeiro, Brazil; g ISGlobal, Hospital Clinic - Universitat de Barcelona, Barcelona, Spain; h Center of Data and Knowledge Integration for Health (CIDACS), Instituto Gonçalo Muniz, Fundação Oswaldo Cruz, Salvador, Brazil; i Comprehensive Health Research Center and Public Health Research Centre, NOVA National School of Public Health, NOVA University of Lisbon, Lisbon, Portugal

**Keywords:** Brazil, Primary care, Hospitalisations, Admissions

## Abstract

**Background:**

Robust evidence on the relationship between primary care and emergency admissions is lacking in low- and middle-income countries. This study evaluates how the phased roll out of the family health strategy (FHS) to the urban poor in Rio de Janeiro Brazil affected emergency hospital admissions and readmissions from ambulatory-care sensitives conditions (ACSCs).

**Methods:**

A cohort of 1.2 million adults in Rio de Janeiro city were followed for five years (Jan 2012 to Dec 2016). The association between FHS use and the likelihood of emergency hospital admissions and 30-day readmissions were evaluated using multi-level Poisson regression models with inverse probability treatment weighting and regression adjustment (IPTW-RA) for socioeconomic and household characteristics. Inequalities in associations were examined across groups of causes and by key socioeconomic groups.

**Results:**

Records from 2,551,934 primary care consultations and 15,627 admissions were analysed. In IPTW-RA analyses, each additional FHS consultation was associated with a 3% lower rate of ACSC admission (RR: 0.97; 95%CI: 0.95, 0.98), a 63% lower rate of 30-day readmissions from any non-birth cause (RR: 0.37; 95%CI: 0.30, 0.46), and an 57% lower rate of 30-day readmissions from ACSCs (RR: 0.43; 95%CI: 0.33, 0.55). Individuals who were older, had the lowest educational attainment, were unemployed, and had higher incomes had larger reductions in ACSC admissions associated with FHS use.

**Interpretation:**

Investment in primary care is important for reducing emergency hospital admissions and their associated costs in LMICs.

**Funding:**

DFID/MRC/Wellcome Trust/ESRC.

**Research in context**

**Evidence before this study**

MEDLINE, via PubMed was searched in February 2022 for studies on primary healthcare and unplanned/emergency admissions. Titles and abstracts were searched using the Mesh terms “Primary Health Care”, “Ambulatory care”, and “Emergency medical Services” and the free word terms primary healthcare and emergency admission, including synonyms and alternative spellings. Studies were restricted to systematic reviews examining impacts and associations due to the size of the evidence base and multiple systematic reviews existing on the topic. Only reviews published after 1999 were included. We included studies examining any population, interventions related to the impact of primary care, and outcomes related to emergency or unplanned admissions. We extracted overall findings from systematic reviews, assessments of the overall quality of the evidence base, impacts on health inequalities, and geographical spread of the evidence.

We identified ten eligible systematic reviews. All exclusively reported evidence from high-income countries, and classified the evidence base as of fair/moderate quality. All reported a role for primary care services in reducing unplanned/emergency admissions, including the importance of accessibility and continuity in primary care and care delivered in the community. Socioeconomic inequalities in the relationship between primary care and admissions were not clearly identified by any systematic review.

**Added value of this study**

This study uses a large cohort and a robust analytical strategy to examine the relationship between primary care utilisation and emergency admissions for ambulatory care sensitive conditions (ACSC) in a low-income population in Rio de Janeiro, Brazil. It finds primary care utilisation is consistently associated with lower rates of ACSC admission and readmission, with evidence of a dose response relationship. Notably, individuals with lower levels of education, who were unemployed, or who had higher incomes disproportionately benefitted by using primary care with greater associated reductions in ACSC admissions compared to those with higher education, who were employed or had lower incomes. By causes of admission, those most reduced by FHS use were infectious gastroenteritis, asthma, lower respiratory diseases, heart failure, cerebrovascular disease, skin infections and female pelvic inflammatory disease.

**Implications of all the available evidence**

Global evidence indicates an important role of primary care in reducing unplanned and emergency admissions to hospital. There is good evidence from high-income countries, and a growing evidence base from low- and middle-income countries such as Brazil. Emerging evidence suggests populations of lower socioeconomic status can benefit more from using primary care. Policymakers must prioritise primary care within health systems as means to reach universal health coverage, diminish costly emergency admissions to hospital, and reduce health inequities.

Alt-text: Unlabelled box

## Introduction

Primary healthcare (PHC) is considered the foundation of efficient, equitable and sustainable high-quality health systems.^1^ Strengthening PHC is central to progressing Universal Health Coverage and advancing towards the Sustainable Development Goals.^2^^,^^3^ Countries with well-developed PHC have better and more equitable health outcomes, and provide stronger financial protection to citizens.^4^

Emergency or unplanned hospital admissions are a major cost for health systems globally. In England, for example, emergency admissions cost an estimated £18 billion in 2019 (out of a total health system budget of £73 billion).^5^ High-quality PHC has the potential to prevent emergency hospital admissions by diagnosing problems early, managing risk factors, and preventing exacerbations.^6^ High-quality, accessible PHC services are associated with fewer emergency admissions – particularly where there is strong continuity of care in PHC.^7^, ^8^, ^9^, ^10^, ^11^, ^12^, ^13^, ^14^, ^15^, ^16^, ^17^ However, in many high-income countries, emergency admissions have increased in recent decades,^8^ including in countries with strong PHC systems such as Denmark,^18^ the UK,^19^^,^^20^ and Portugal.^8^ The convenience of emergency care, ageing populations with chronic conditions, socioeconomic deprivation, limited PHC (especially out of hours services), and increasing use of day-case interventions (with complications requiring admissions) may explain increasing emergency admissions seen in many countries.^8^ In low- and middle-income countries (LMICs), there is very little robust evidence on the impact of PHC on emergency hospital activity, including inequalities in any impacts – an important evidence gap constraining policy making.

Brazil is a valuable setting for evaluating PHC and contributes significantly to the evidence base of PHC.^21^ The country has expanded its PHC system substantially over recent decades, investing in comprehensive, multi-disciplinary, community-based services,^22^ and has high-quality data for analysis.^23^ This has been through the Family Health Strategy (FHS) - a multidisciplinary model of PHC including doctors, nurses and community health workers covering approximately 1000 local families and providing a range of services including acute care, referral, risk factor management, prevention, health promotion, and health education, and home visits.^22^ However, urban areas in Brazil have historically had lower coverage with PHC that rural areas.^24^ Evidence demonstrates that PHC in Brazil has contributed to improvements in health and reductions in health inequalities,^24^, ^25^, ^26^, ^27^, ^28^, ^29^, ^30^ but evidence gaps remain. Much evidence on PHC and admissions in Brazil comes from ecological studies with limited attention to inequalities. Furthermore, despite a wealth of studies, there is no consensus on the relationship between PHC and emergency admissions in Brazil.^25^^,^^26^^,^^28^^,^^31^ On one hand, admissions may increase if PHC facilitates access to secondary care, there is unmet demand, or healthcare issues cannot be resolved in PHC.^32^ Conversely, high quality PHC can resolve many needs locally, and through prevention and promotion reduce future hospitalisations or readmissions.

In Rio de Janeiro, PHC, through the FHS, was substantially expanded beginning in 2008, and was prioritised in poor areas lacking services. By 2016, over 50% of the population in the city were covered. This study evaluates the expansion of PHC in the city of Rio de Janeiro aiming to explore associations between FHS usage and emergency ambulatory care sensitive condition (ACSC) hospital admissions and readmissions.

## Methods

### Study design

This is a cohort study following 1.2 million adults (aged 15-84 years) applying for government welfare in the city of Rio de Janeiro, Brazil between 1st Jan 2010 and 31st Dec 2016. FHS use and hospital use were ascertained through comprehensive linkage of records.^33^ Individuals’ risk of admission and associations with FHS use were assessed using multiple regression. Doubly-robust inverse probability treatment weighting and regression adjustment (IPTW-RA) was used to reduce bias from potential non-random FHS coverage and increase causal inference.^34^

### Data sources

The cohort was built from three datasets:^33^ i) the *Cadastro Único* (the unified registry) – a national database of families registering to claim welfare that includes in-depth demographic and socioeconomic data (the study population); ii) FHS Electronic health records (EHR) - a municipal database containing individuals’ registration with FHS and their utilisation records; and iii) the *Sistema de Informações Hospitalares* (Hospitalisation Information System; SIH) - a national database of all hospital records for admissions funded by the public health system. These were obtained from the Rio de Janeiro *Secretaria Municipal de Saúde* (Municipal Health Secretariat) and *Secretaria Municipal de Assistência Social* (Municipal Secretariat of Social Assistance; SMAS).

The three datasets were linked via a combination of deterministic and probabilistic approaches. This involved matching name, date of birth and tax numbers using deterministic linkage, phonetic name matching with Levenshtein distance matching, followed by manual review. Methods describing the linkage are published elsewhere.^24^^,^^33^

The study population covers approximately 25% of the city population of 6.7 million. Given *Cadastro Único* contains individuals attempting to claim welfare, the population are predominantly of lower income. Duplicate records, those with invalid *Cadastro Único* registration throughout the period, and those erroneously registered in the *Cadastro Único* after their death were excluded.

### Database structure

The linked datasets were combined into a multilevel structure – multiple distinct time-period observations clustered per individual. These were distinct observations per calendar year under observation (Jan 1 to Dec 31) allowing time-varying effects to be modelled. The number of observations varied as individuals had different entry dates to the *Cadastro Único* cohort. For individuals who were hospitalised, annual time observations were split at the date of each hospitalisation to model the risk of each hospitalisation separately (rather than annual counts). These multiple observations were adjusted by an individual's person-years under observation to remove any biases from multiple observations (see below).

The study population included 1,240,009 adults. This was generated from 1,762,905 individuals present in the *Cadastro Único* living in the city of Rio de Janeiro, after excluding duplicate records (*n=*83,583), those under 15 years on 31^st^ Dec 2016 (*n=*424,243), those 85 years or older on 1st Jan 2010 (*n=*2218), records where individuals died before 1^st^ Jan 2010 (*n=*12397), and erroneous records where individuals were hospitalised after death (*n=*455).

### Variables

The primary outcomes were emergency (unplanned) hospital admissions and readmissions. Ambulatory care sensitive conditions (ACSCs) were studied for admission and readmissions. Readmission was defined as an admission to hospital within 30-days of a hospital ACSC previous admission. ACSCs are defined “as those health conditions for which hospitalisations can be avoided by timely and effective care in ambulatory [primary care] settings”.^35^ Only emergency admissions were analysed in line with international definitions of ACSCs,^35^ to reflect the costly and clinically-intense burdens on hospitals, and capture unmanaged and untreated health conditions. Admissions from ACSCs were defined based on primary and secondary diagnosis codes (ICD10 codes) devised by the Brazilian Ministry of Health (Table S1 supplementary material).^36^ Secondary outcomes were hospital admissions by 19 subgroups of ACSCs.

The main exposure variable was FHS use. It was the number (count) of doctor or nurse FHS consultations for each time period observation. This was either the calendar year observation for each individual, or for individuals with admissions (whose time period observation were further split at each admission), the time period before admission.

Covariates employed in all regression models were: sex (male; female); self-declared race/ethnicity (White; Black; pardo/mixed race; or other - including Asian, Indigenous or not declared); age group on cohort entry (15–19 years; 20–24; 25–29 30–34; 35–39; 40–44; 45–49; 50–59; 60–69; and 70+); highest educational attainment (preschool/literacy class/none; elementary school; high school; or higher education); self-reported disability (yes; no); unemployed (yes; no); per capita household income quintiles (Q1 less than R$45 (USD$8); Q2 R$45–74 (USD$8–13); Q3 R$75–114 (USD$14–20); Q4 R$114–197 (USD$21–35); R$197 (USD$35) or more); number of children in household (none; one; two; three of more); per capita household expenditure on medicines (None; 0-R$50 (USD$0–9); more than R$50 (USD$9)); if there was formal employment within the family (yes; no); and if the family was in receipt of Brazil's conditional cash transfer social welfare (*Bolsa Família*).

Additional variables included in the models for generating Inverse Probability of Treatment Weighting (IPTW) were: number of family members per bedroom (two or fewer, two-three, three-four, four or more); family size (one, two, three, four, five, six or more); household flooring (cement, wood, ceramic or tiles, or other); household piped water access (yes or no); if an individual had formal labour employment (yes; no); quintile of per capita household expenditure on food; and if an individual was registered seven days or more before first FHS use. Collinearity was checked with VIFs (Variance Inflation Factor).

### Analyses

Individual-level weights for IPTW were generated. Adjusted logistic regression models employing all covariates and additional IPTW variables were used to model and predict individuals’ probabilities of FHS use.^34^ This was to weight FHS users and non-users to balance observed covariates aiming to reduce potential bias from the prioritization of FHS implementation in the most vulnerable areas (i.e. non-random FHS roll out).^34^

Multi-level modelling approaches were used to account for the clustered nature of the data (multiple time observations per individual). Models were adjusted for all covariates specified above and employed individual-level random intercepts. No aggregate/higher-level variables were used. Robust standard errors were clustered at the individual level to deal with potential model misspecification and account for the clustered nature of the data.

The associations between FHS use, hospital admission and readmission were assessed through regression models. Poisson regression models were used to model the outcomes (a binary specification denoting admission in that person-time observation) and included an offset term to capture an individual's observation time (for each time observation – for example one person-year for a whole calendar year observation). The total observation time per individual was the time from cohort entry to cohort exit (either 31st Dec 2016 or date of death) and is the sum of all time observations per individual. This approach adjusts for differences in individuals’ periods of observation (and risk of admission), accounts for time-varying nature of FHS use and hospital admission, and is an approach frequently used before.^37^, ^38^, ^39^ Model coefficients were expressed as rate ratios (RR). The RR for FHS use is interpreted as the admission rate (admissions per person-years) for one FHS consultation divided by the admission rate for no FHS use.

The ordering of the analyses was as follows. First, data on the cohort were presented descriptively. Second, adjusted multi-level Poisson regression models with IPTW were carried out for the primary outcomes (admissions and 30-day readmissions) to assess the association between FHS use and admissions. Third, interactions were used to explore the heterogeneous associations (for admissions only due to small numbers of readmissions) with FHS use. The selection of variables for heterogeneity analysis was based on key socioeconomic groups identified in wider literature. The regression models above were repeated for each key socioeconomic variable (sex, race, age, education, employment status, income quintile, Bolsa Familia recipient status and formal employment status), but with an interaction between FHS use and the socioeconomic of interest. Coefficients for relative associations with FHS use across models were compared graphically, and predicted ACSC admission rates by each socioeconomic group were plotted under FHS use and no FHS use scenarios. Fourth, the association between FHS use and secondary outcomes (causes of admission) were explored with the same regression models specified above.

### Robustness checks

To test for potential biases from unobserved confounding (i.e. health status) associated both with first FHS use and admission, the time periods 90 days before and after first FHS use were identified and excluded from the analysis (including any admissions) and regression models repeated. Additionally, the models were repeated for elective admissions from ACSCs to examine impacts on non-emergency care. Models on primary outcomes were also repeated without IPTW to examine potential biases from weighting. To examine potential biases from utilisation of FHS by healthy women during pregnancy, we repeated the models excluding FHS consultations relating to healthy, normal pregnancy (ICD10 codes Z32-Z34, Z36; and International Classification of Primary Care (ICPC-2) produced codes W01 and W78).

### Ethical approval

Approval for this study was obtained from Imperial College London and the Brazilian National Commission for Ethics in Research (*Comissão Nacional de Ética em Pesquisa* (CONEP)) – number 2.689.528.

The authors had full access to all anonymised databases employed in this analysis. Identifiable datasets for linkage were securely held by co-author (C Medina Coeli) for carrying out linkages and the generation of linkage keys to link the anonymised datasets.

### Role of the funding source

This study was supported by the UK's Joint Health Systems Research Initiative (DFID/MRC/Wellcome Trust/ESRC) grant number MR/P014593/1. This funder had no role in the study design, in the collection, analysis, and interpretation of data, in the writing of the report, or in the decision to submit the paper for publication.

## Results

A total of 1,240,009 adults (aged 15–84 years at any point between 1 Jan 2010 to 31 Dec 2016) were included in the study accounting for 6,495,642 person years (Table 1). A total of 2,551,934 primary care consultations and 15,627 linked emergency admissions from ACSCs were analysed. For readmissions within 30 days of a prior ACSC admission, there were 796 readmissions for any non-birth cause and 484 readmissions for ACSCs. By the end of the cohort period, 446,567 adults (37.6%) had used FHS services at least once and were considered FHS users, while 1.1% of individuals (13,352) had at least one emergency admission and 0.06% (777) has at least one emergency readmission (for any non-birth cause following prior ACSC admission).

**Table 1:** Socioeconomic and demographic characteristics of the study population.

	N			% (unweighted)		% (weighted)	
	Non-users	FHS users	Total	Non-users	FHS users	Non-users	FHS users
Sex							
Male	349,575	139,371	488,946	71.5	28.5	50.2	49.8
Female	423,867	327,196	751,063	56.4	43.6	50.1	50.0
Race							
White	231,067	135,505	366,572	63.0	37.0	50.1	49.9
Black	133,096	83,254	216,350	61.5	38.5	50.1	49.9
Parda	390,391	238,780	629,171	62.1	38.0	50.1	49.9
Other	18,888	9,028	27,916	67.7	32.3	50.2	49.8
Age (years)							
15–19	142,056	75,580	217,636	65.3	34.7	49.9	50.1
20–24	126,810	62,946	189,756	66.8	33.2	50.3	49.7
25–29	89,657	45,212	134,869	66.5	33.5	50.3	49.7
30–34	71,460	41,626	113,086	63.2	36.8	50.3	49.7
35–39	71,460	43,897	115,357	62.0	38.1	50.3	49.8
40–44	64,689	41,303	105,992	61.0	39.0	50.1	49.9
45–49	55,366	37,399	92,765	59.7	40.3	50.1	50.0
50–59	80,967	62,316	143,283	56.5	43.5	50.0	50.1
60–69	43,865	38,571	82,436	53.2	46.8	49.8	50.2
70+	27,112	17,717	44,829	60.5	39.5	50.0	50.0
Education level							
Preschool/Literacy class/None	67,828	34,967	102,795	66.0	34.0	50.3	49.8
Elementary school	465,811	291,257	757,068	61.5	38.5	50.2	49.8
High school	225,956	135,489	361,445	62.5	37.5	50.0	50.1
Higher education	13,847	4,854	18,701	74.0	26.0	50.6	49.4
Disability							
No	749,016	444,361	1,193,377	62.8	37.2	50.1	49.9
Yes	24,426	22,206	46,632	52.4	47.6	49.8	50.2
Unemployed							
No	592,123	323,246	915,369	64.7	35.3	50.2	49.8
Yes	181,319	143,321	324,640	55.9	44.2	50.0	50.0
Household characteristics							
Income Quintiles							
Q1 (<R$45; poorest)	146,980	82,257	229,237	64.1	35.9	50.3	49.7
Q2 (R$$45–74)	141,305	90,190	231,495	61.0	39.0	50.2	49.8
Q3 (R$75–114)	150,923	92,783	243,706	61.9	38.1	50.1	49.9
Q4 (R$114–197)	154,492	96,418	250,910	61.6	38.4	50.0	50.0
Q5 (R$197+; richest)	179,742	104,919	284,661	63.1	36.9	50.0	50.0
Number of children in family							
None	410,064	229,152	639,216	64.2	35.9	50.1	49.9
One	219,884	138,172	358,056	61.4	38.6	50.1	49.9
Two	98,682	67,224	165,906	59.5	40.5	50.1	49.9
Three of more	44,812	32,019	76,831	58.3	41.7	50.2	49.8
Bolsa Familia claiming family?							
No	278,670	134,845	413,515	67.4	32.6	50.3	49.7
Yes	494,772	331,722	826,494	59.9	40.1	50.0	50.0
Per capita medicine expenditure							
None	624,699	368,569	993,268	62.9	37.1	50.1	49.9
0-R$50	92,531	63,368	155,899	59.4	40.7	50.1	49.9
>R$50	56,212	34,630	90,842	61.9	38.1	50.0	50.0
Formal employment in family							
No	606,702	366,789	973,491	62.3	37.7	50.1	49.9
Yes	166,740	99,778	266,518	62.6	37.4	50.0	50.0
Individuals ever admitted for ACSC	7,427	5,925	13,352	55.6	44.4	45.1	54.9
Individuals ever readmitted for ACSC following prior ACSC admission	252	175	426	59.1	40.9	48.2	51.8
Individuals ever readmitted for any non-birth cause following prior ACSC admission	445	254	699	63.7	36.3	54.2	45.8
Total individuals	773,442	466,567	1,240,009	62.4	37.6	50.1	49.9
Person-Years of observation	4,008,545	2,487,096	6,495,642	-	-	-	-

In adjusted multi-level Poisson regression models, both hospital admissions and 30-day readmissions were heavily socially patterned with higher admission rates for individuals who were black, older, of lower educational attainment, disabled, poorer, in receipt of welfare (Bolsa Familia), having any household expenditures on medicines, or without formal employment in the household (Table 2). An increase of one FHS consultation was associated with a 3% lower rate of ACSC admission (RR: 0.97; 95%CI: 0.95, 0.98), a 63% lower rate of 30-day readmissions from any non-birth cause (RR: 0.37; 95%CI: 0.30, 0.46), and an 57% lower rate of 30-day readmissions from ACSCs (RR: 0.43; 95%CI: 0.33, 0.55). This translated into an absolute reduction of 6.7 admissions per 100,000 person years (a modelled rate of 185.5 ACSC admissions per 100,000 if all individuals had one FHS consultations compared to 192.1 if no one used FHS), a reduction of 20.3 any-cause readmission per 100,000 (a rate of 11.9 for one FHS consultation and a rate of 32.2 for no FHS use) and a reduction of 5.8 ACSC readmissions per 100,000 person years (a rate of 4.3 for one FHS consultation and a rate of 10.1 for no FHS use). Increasing FHS use, measured categorically, was generally associated larger reductions in ACSC admission rates (Table S3 supplementary material). For example, compared to those with no FHS use, individuals with four or five FHS consultation had a 18% lower ACSC admission rate (RR: 0.82; 0.71, 0.94), whilst six to nine FHS consultations had a 35% lower ACSC admission rate (RR: 0.65; 95%CI: 0.54, 0.778). However, only one FHS consultation was associated with a 23% increased rate of ACSC admissions (RR: 1.23; 95%CI: 0.14, 0.33) compared to no FHS use.

**Table 2:** Results from multilevel Poisson models on ACSC admissions and 30-day readmissions.

	ACSC Admissions		30-day readmission (any nonbirth cause)		30-day readmission (ACSC only)	
	RR	95%CI	RR	95%CI	RR	95%CI
FHS consultations	0.965⁎⁎⁎	0.951,0.980	0.369⁎⁎⁎	0.297,0.459	0.426⁎⁎⁎	0.329,0.553
Sex						
Male	1 (Ref)		1 (Ref)		1 (Ref)	
Female	1.192⁎⁎⁎	1.140,1.246	0.722⁎⁎	0.593,0.877	1.096	0.852,1.410
Race						
White	1 (Ref)		1 (Ref)		1 (Ref)	
Black	1.275⁎⁎⁎	1.205,1.350	1.483⁎⁎	1.143,1.924	1.481*	1.096,2.001
Parda	1.059*	1.011,1.111	1.031	0.807,1.316	0.812	0.618,1.066
Other	1.097	0.950,1.268	1.190	0.689,2.056	1.056	0.497,2.245
Age (years)						
15–19	1 (Ref)		1 (Ref)		1 (Ref)	
20–24	2.128⁎⁎⁎	1.912,2.370	2.144	0.512,8.973	1.324	0.728,2.409
25–29	2.033⁎⁎⁎	1.811,2.282	1.515	0.346,6.633	0.959	0.479,1.921
30–34	1.636⁎⁎⁎	1.448,1.849	3.163	0.690,14.503	1.047	0.530,2.067
35–39	1.553⁎⁎⁎	1.369,1.761	4.843*	1.091,21.503	0.701	0.347,1.417
40–44	1.733⁎⁎⁎	1.525,1.971	6.724*	1.563,28.917	0.824	0.382,1.775
45–49	2.472⁎⁎⁎	2.183,2.798	14.898⁎⁎⁎	3.537,62.748	1.820	0.920,3.603
50–59	3.847⁎⁎⁎	3.449,4.291	23.093⁎⁎⁎	5.834,91.406	2.984⁎⁎⁎	1.580,5.636
60–69	7.145⁎⁎⁎	6.394,7.985	64.883⁎⁎⁎	16.700,252.077	9.150⁎⁎⁎	4.839,17.302
70+	14.517⁎⁎⁎	12.884,16.356	97.557⁎⁎⁎	25.109,379.046	14.302⁎⁎⁎	7.282,28.087
Education level						
Preschool/Literacy/None	1 (Ref)		1 (Ref)		1 (Ref)	
Elementary school	0.901⁎⁎	0.844,0.961	1.01	0.769,1.326	0.876	0.603,1.271
High school	0.634⁎⁎⁎	0.587,0.685	0.524⁎⁎	0.339,0.809	0.468⁎⁎	0.296,0.741
Higher education	0.446⁎⁎⁎	0.334,0.595	0.451	0.159,1.275	0.075*	0.010,0.547
Disability						
No	1 (Ref)		1 (Ref)		1 (Ref)	
Yes	2.162⁎⁎⁎	2.015,2.320	2.264⁎⁎⁎	1.785,2.872	1.802⁎⁎	1.241,2.615
Unemployed						
No	1 (Ref)		1 (Ref)		1 (Ref)	
Yes	1.120⁎⁎⁎	1.069,1.173	1.933⁎⁎⁎	1.575,2.372	1.946⁎⁎⁎	1.487,2.547
Income Quintiles						
Q1 (<R$45; poorest)	1 (Ref)		1 (Ref)		1 (Ref)	
Q2 (R$$45–74)	0.870⁎⁎⁎	0.816,0.928	1.257	0.878,1.797	0.906	0.609,1.349
Q3 (R$75–114)	0.825⁎⁎⁎	0.773,0.880	0.836	0.592,1.178	0.986	0.667,1.458
Q4 (R$114–197)	0.776⁎⁎⁎	0.726,0.830	1.065	0.776,1.463	0.799	0.525,1.215
Q5 (R$197+; richest)	0.716⁎⁎⁎	0.664,0.771	0.985	0.723,1.344	0.893	0.598,1.335
Number of children in family						
None	1 (Ref)		1 (Ref)		1 (Ref)	
One	0.987	0.936,1.041	1.103	0.838,1.453	1.229	0.902,1.673
Two	1.199⁎⁎⁎	1.123,1.280	1.116	0.807,1.543	1.403	0.960,2.051
Three of more	1.446⁎⁎⁎	1.335,1.566	1.273	0.819,1.979	1.256	0.755,2.090
Bolsa Familia claiming family?						
No	1 (Ref)		1 (Ref)		1 (Ref)	
Yes	1.257⁎⁎⁎	1.192,1.325	1.327*	1.064,1.654	1.303	0.973,1.744
Per capita medicine expenditure						
None	1 (Ref)		1 (Ref)		1 (Ref)	
0-R$50	1.082⁎⁎	1.021,1.146	0.868	0.673,1.119	0.842	0.604,1.174
>R$50	1.194⁎⁎⁎	1.116,1.278	1.003	0.781,1.289	0.715	0.475,1.077
Formal employment in family						
No	1 (Ref)		1 (Ref)		1 (Ref)	
Yes	0.910⁎⁎	0.859,0.963	0.984	0.760,1.275	0.828	0.588,1.165
Total individuals	1240009		1240009		1240009	

Results from adjusted multilevel Poisson regression models. Models additionally adjusted for time effects and include a person-year offset.

⁎ *p*<0.05.

⁎⁎ *p*<0.01.

⁎⁎⁎ *p*<0.001.

**Figure 1:**
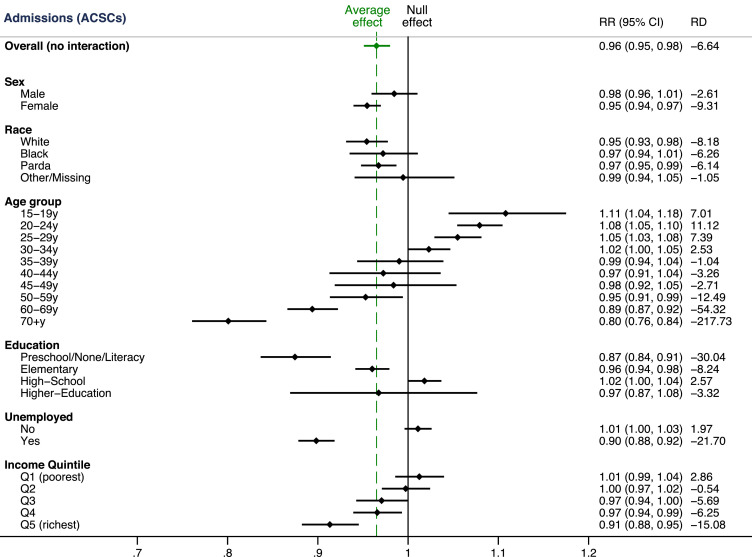
**Inequalities in the association between FHS consultations and ACSC admissions**. ACSC – Ambulatory care sensitive condition; RR – Rate Ratio; RD – Rate difference; 95% CI – 95% Confidence Interval.

**Figure 2:**
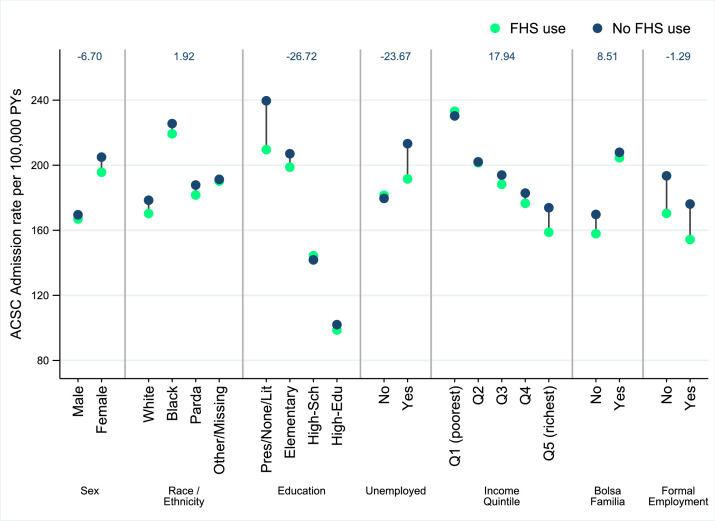
**Predicted ACSC admission rates comparing one FHS consultation and no FHS use across socioeconomic groups.** Coefficients for each socioeconomic group taken from separate regression models with interactions between FHS consultations and socioeconomic grouping variable (i.e., sex). Predicted ACSC admission rates obtained from post-regression prediction under one FHS consultation and FHS usage scenarios for the socioeconomic group of interest. All other variables (including socioeconomic factors) held constant. Change in within-group inequality calculated as the difference in the within group inequality (i.e., the range of admission rates within a group) from no FHS to FHS use. A negative value indicates reduced within-group inequality. ACSC – Ambulatory care sensitive condition; FHS – Family Health strategy.

Interactions were used to document the heterogeneous associations with FHS use across socioeconomic subgroups (Figure 1). Increasing FHS use was associated with a lower rate of admissions from ACSCs = except for those aged under 35 years of age. There were larger relative and absolute (i.e., rate differences) reductions in ACSC admissions for individuals who were female, who were older, who had the lowest educational attainment, who were unemployed, and who were in higher income groups. This translated into overall reductions in within-group inequalities in ACSC admission rates for some socioeconomic groups (sex, education and employment groups), but there were other socioeconomic groupings (by income and Bolsa Familia recipient status) where within-group inequalities increased (Figure 2).

**Figure 3:**
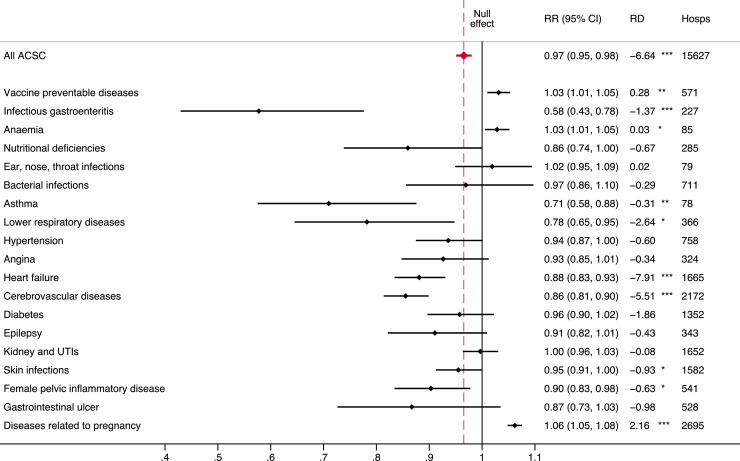
**Association between FHS use and ACSC admissions by groups of causes.** ACSC – Ambulatory care sensitive condition; RR – Rate Ratio; RD – Rate difference; UTI – Urinary tract infection; 95% CI – 95% Confidence Interval; * *p*<0.05; ^⁎⁎^*p*<0.01; ^⁎⁎⁎^*p*<0.001.

The leading cause of ACSC admissions (by ICD10 category) were infections of the genitourinary tract in pregnancy (16.5%), stroke (10.3%), heart failure (9.1%), disorders of urinary system (6.6%), and cellulitis (659 admissions; 4.2%) (Table S2 supplementary material). There were heterogenous associations between FHS use and ACSC admissions by groups of causes (Figure 3). FHS use was generally associated with lower admission rates for most groups of causes, but effect sizes were imprecisely estimated and some non-significant due to small numbers. An increase of one FHS consultation, however, was significantly associated with a 42% lower admissions rate from infectious gastroenteritis (RR: 0.58; 95%CI: 0.43, 0.78), a 29% lower admissions rate from asthma (RR: 0.71; 95%CI: 0.58, 0.88), a 22% lower admissions rate from lower respiratory diseases (mainly chronic obstructive pulmonary disorder (COPD); RR: 0.78; 95%CI: 0.65, 0.95), a 12% lower rate from heart failure (RR: 0.88; 95%CI: 0.83, 0.93), a 14% lower rate from cerebrovascular disease (∼75% stroke; RR: 0.86; 95%CI: 0.80, 0.90), a 5% lower rate from skin infections (RR: 0.95; 95%CI: 0.91, 1.00), and a 10% lower rate from female pelvic inflammatory disease (RR: 0.90; 95%CI: 0.83, 0.98). Increasing FHS use was associated with a 3% increase in the admission rate for vaccine preventable diseases (>75% tuberculosis; RR: 1.03; 95%CI: 1.01, 1.05), a 3% increase in the rate for anaemia (90% iron deficiency anaemia; RR: 1.03; 95%CI: 1.01, 1.05), a 6% increase in the admission rate for diseases related to pregnancy (96% infections of the genitourinary tract; RR: 1.06; 95%CI: 1.05, 1.08).

### Robustness checks

Excluding the 90 days of observation both before and after first FHS use and repeating the analyses showed concordance in results suggesting potential biases from FHS use and unobserved health status were likely to be minimal. In these analyses, increases in FHS use was associated with a 4% lower rate of ACSC admission (RR: 0.96; 95%CI: 0.95, 0.98), a 65% lower rate of any-cause 30-day readmissions (RR: 0.35; 95%CI: 0.31, 0.40), and 60% lower rate of 30-day readmissions from ACSCs (RR: 0.40; 95%CI: 0.30, 0.53) (Table S4 supplementary material). When examining elective admissions from ACSCs, there were similar associated reductions in admissions associated with FHS use (RR: 0.96; 95%CI: 0.94 0.99) (Table S5 supplementary material). Models without the IPTW showing similar effect sizes (for ACSC admissions RR: 0.94; 95%CI: 0.93, 0.96) to the IPTW analysis suggesting unweighting had not introduced significant bias (Table S6 supplementary material). Removing FHS consultations relating to healthy pregnancy also showed very similar effect sizes (for ACSC admissions RR: 0.96; 95%CI: 0.94, 0.97) (Table S7 supplementary material).

## Discussion

Using a cohort of 1.2 million low-income individuals in Rio de Janeiro with linked primary care and hospital records, FHS utilisation was found to be associated with a lower likelihood of emergency admission from ACSCs and substantially lower likelihood of readmission within 30 days of a previous ACSC admission. There were mixed findings relating to inequalities, as some more deprived socioeconomic groups experienced larger reductions in ACSC admissions associated with FHS use (e.g. those of lower education and the unemployed), but those of higher incomes also benefitted disproportionately more than lower income individuals.

The finding that community-based PHC services are associated with reductions in emergency admission is unsurprising given primary care's focus on managing chronic diseases, treating acute conditions, and providing preventative and health promoting services. International evidence indicates accessible primary care, and a good supply of primary care physicians are important for reducing ACSC admissions, although most of this knowledge comes from high-income countries.^7^, ^8^, ^9^, ^10^, ^11^, ^12^, ^13^, ^14^, ^15^, ^16^, ^17^ The findings from this study are also concordant with evidence on mortality from the same cohort.^24^ However, the evidence base from Brazil on the association between PHC and emergency admissions is mixed.^25^^,^^26^^,^^28^^,^^31^ In Rio de Janeiro, the FHS may be associated with reductions in ACSC admissions due to investments in primary care services and a focus on improving access and quality by building new clinics near to communities to be covered (i.e. in or near favelas), contracting through non-governmental organisations to increase physician remuneration, a dedicated residency programme on family medicine, and comprehensive services including X-rays, ultrasound, and minor surgery.^40^, ^41^, ^42^ There was acknowledgement during expansion of the FHS in Rio de Janeiro, that without high-quality comprehensive services patients may choose to go directly to hospitals.^40^^,^^43^ In 2012, the national Program for Improving Access and Quality in Primary Care (PMAQ) evaluated 65% of the teams in Rio de Janeiro as “good” or “excellent”.^44^

There was a finding that some more deprived socioeconomic groups and those with larger underlying health needs^45^ had larger relative reductions in ACSC admissions. This included those with the lowest educational attainment and the unemployed. These populations have poorer health outcomes and lower healthcare use and are likely to be burdened with worse social determinants of health.^46^, ^47^, ^48^ Older individuals also had larger relative reductions in ACSC admission rates, which is unsurprising given the association between age and poorer health. These findings may be indicative of PHC's accessibility and ability to provide care to those with greater health needs.^49^ Despite having the lowest rates of ACSC admissions, individuals of the highest income quintile also had larger relative reductions in ACSCs admissions (than other income quintiles) which is contrary to patterns in other socioeconomic groups. It is important to note, income quintiles are relative to the *Cadastro Único* study population, which is mainly lower income families claiming welfare, the highest income quintiles in this population may be at the lower-to-middle range of incomes across the wider city. These individuals may have some access to private health insurance, increased abilities to purchase medicines, and greater health-seeking behaviours, potentially explaining the results. This could also be explained by persisting financial barriers to accessing primary care, such as forgone employment, or poorer health literacy and treatment adherence for the lowest income populations.

An increase of one FHS consultation, however, was significantly associated with a 42% lower admissions rate from infectious gastroenteritis (RR: 0.58; 95%CI: 0.43, 0.78), a 29% lower admissions rate from asthma (RR: 0.71; 95%CI: 0.58, 0.88), a 22% lower admissions rate from lower respiratory diseases (mainly chronic obstructive pulmonary disorder (COPD); RR: 0.78; 95%CI: 0.65, 0.95), a 12% lower rate from heart failure (RR: 0.88; 95%CI: 0.83, 0.93), a 14% lower rate from cerebrovascular disease (∼75% stroke; RR: 0.86; 95%CI: 0.80, 0.90), a 5% lower rate from skin infections (RR: 0.95; 95%CI: 0.91, 1.00), and a 10% lower rate from female pelvic inflammatory disease (RR: 0.90; 95%CI: 0.83, 0.98). Increasing FHS use was associated with a 3% increase in the admission rate for vaccine preventable diseases (>75% tuberculosis; RR: 1.03; 95%CI: 1.01, 1.05), a 3% increase in the rate for anaemia (90% iron deficiency anaemia; RR: 1.03; 95%CI: 1.01, 1.05), a 6% increase in the admission rate for diseases related to pregnancy (96% infections of the genitourinary tract; RR: 1.06; 95%CI: 1.05, 1.08).

The finding that infectious gastroenteritis, asthma, lower respiratory diseases, heart failure, cerebrovascular diseases, skin infections, and female pelvic inflammatory disease were ACSCs most sensitive to FHS use is plausibly explained by the preventative and promotive actions of PHC. For example, infectious gastroenteritis, skin infections, and infections related to female pelvic inflammatory disease can often be treated with antibiotics in PHC, reducing risk of complications and admission. Emergency admissions for COPD and emphysema (∼75% of ACSC admissions for lower respiratory diseases in this study) can be prevented with pharmacotherapies, pulmonary rehabilitation, and smoking cessation – all interventions that be managed from PHC.^50^ Similarly, hypertension and behavioural risk factors (which affect the risk of heart disease and stroke) can be managed in PHC, reducing the likelihood of emergency admissions. It is notable that for some conditions, there were increases in the likelihood of ACSC admission associated with FHS use, specifically tuberculosis, anaemia, and infections of the genitourinary tract. This could be due to PHC's role in identifying advanced cases and expediting necessary hospital admission.

This study has important limitations. Firstly, only publicly funded emergency (unplanned) admissions from ACSCs are studied, without analysis of other admission types and causes. There could be concomitant increases in other causes, however there were also reductions in elective hospital care associated with FHS use. Individuals could utilise private hospital care, but given the low-income focus of the study population this is likely to be low. Unfortunately, data on health insurance or private healthcare use was not available, which may have explained some of the findings for the highest income quintile of the population. Secondly, the analysis relies on administrative datasets which could also have introduced bias. For example, if incorrect linkage patterns were associated with admissions or FHS use it could result in skewed associations. However, an evaluation of the linkage process showed a precision of 99%.^33^ Thirdly, unobserved differences between FHS using and non-using individuals may exist. Whilst, IPTW-RA was used to balance all observed covariates and minimize the potential and impact of unobserved confounding,^34^ there is potential for residual confounding. Fourthly, the expansion of the FHS model in Rio was based on investment in new clinics and equipment, as well as a residency programme for family physicians, that may limit the generalisability of these findings to less well-resourced settings. Fifthly, wider health system factors (such as changes in hospital service provision) could potential contribute to changes in utilisation patterns biasing these findings, however there is no evidence of major changes in hospital provision or the location of deaths (which may be suggestive of forgone hospital care; Supplementary Tables S8 and S9).

Key strengths of this study including the use of an extensive and large dataset of individual records with linked health and healthcare data, substantial statistical power to examine socioeconomic inequalities, and the use of IPTW-RA as one of the strongest methods to minimize potential unobserved biases.^34^ Notably, a dose response relationship between increasing FHS use and reductions in emergency admissions was identified strengthening confidence in the plausibility of the findings.

Health systems with free and accessible emergency care, and weak PHC may encourage greater use of emergency hospital care by patients, even in the absence of clinical need^8^ – a common issue for many countries, including Brazil. Policymakers should note that accessible and high-quality primary care can reduce emergency admissions, in addition to reducing morbidity and health inequities. This has important implications for health system sustainability given the high healthcare costs from emergency admissions. Furthermore, these results were found in a poor urban environment, suggesting similar benefits could be accrued in other LMICs covering lower-income populations.

## Conclusions

Increasing coverage of FHS in the city of Rio de Janeiro between 2010 and 2016 was associated with reductions in emergency admissions and readmission from ACSCs. There is some evidence that low educated, unemployed and higher-income individuals experienced greater benefits from FHS use. Primary care is an essential service for strengthening health systems, improving health, reducing inequities and managing healthcare costs.

## Contributors

This study was conceived by BD, CM, TH and DR. Data acquisition and linkage were undertaken by VS and CMC. TH undertook the analyses with inputs from AT, RP, JM and CM. TH wrote the first draft of the paper with inputs, revisions and edits from all authors.

## Data sharing statement

The datasets generated and analysed during the current study are not publicly available due confidentiality of the linked data. They are available from the corresponding author on reasonable request and following approval from the Brazilian National Commission for Ethics in Research (Comissão Nacional de Ética em Pesquisa (CONEP)) and the Municipal government of Rio de Janeiro.

## Funding

DFID/MRC/Wellcome Trust/ESRC.

## Declaration of interests

BD was Undersecretary of Health Promotion, Surveillance, and Primary Care at the Secretaria Municipal de Saúde, Rio de Janeiro when this project was conceived. VS is a Coordinator of Health Situation Analysis in the Health Surveillance Department, at the Secretaria Municipal de Saúde, Rio de Janeiro. All other authors declare they have no competing interests.

## Appendix Supplementary materials

Image, application 1
