# Supplementary material for: Expansion of primary healthcare and emergency hospital admissions among the urban poor in Rio de Janeiro Brazil: A cohort analysis
Source: Lancet Reg Health Am. 2022 Sep 5;15:100363. doi: 10.1016/j.lana.2022.100363 (PMC9904151; doi:10.1016/j.lana.2022.100363)
Supplement: Supplementary file 1 [file mmc1.docx]

**SUPPLEMENTARY MATERIAL**

**Table S1 - Ambulatory care sensitive conditions (ACSC) with International Classification of Disease (ICD-10) codes**

| **Groups of Conditions** | **ICD-10 Codes** |
| --- | --- |
| Vaccine preventable diseases | A15-A16, A17, A18, A19, A33-A35, A36, A37, A51-A53, A95, B05, B06, B16, B26, B50-B54, B77, I00-I02, G00.0 |
| Infectious gastroenteritis | A00-A09, E86 |
| Anaemia | D50 |
| Nutritional deficiencies | E40-E46, E50-E64 |
| Ear, nose, and throat infections | H66, J00-J03, J06, J31 |
| Bacterial infections | J13-J14, J15.3-J15.4, J15.8-J15.9, J18.1 |
| Asthma | J45-J46 |
| Diseases of the lower respiratory tract | J20, J21, J40-J42, J43, J44, J57 |
| Hypertension | I10-I11 |
| Angina | I10 |
| Heart failure | I50, J81 |
| Cerebrovascular disease | I63-I67, I69, G45-G46 |
| Diabetes mellitus | E10-E14 |
| Epilepsy | G40-G41 |
| Infections of the kidney and urinary tract | N10-N12, N30, N34, N39.0 |
| Infections of the skin and subcutaneous tissue | A46, L01-L04, L08 |
| Female pelvic inflammatory disease | N70-N73, N75-N76 |
| Gastric Ulcers | K25-K28, K92.0, K92.1, K92.2 |
| Diseases related to pregnancy | P35.0, O23, A50 |
|  |  |

Source: Alfradique ME, Bonolo Pde F, Dourado I, Lima-Costa MF, Macinko J, Mendonca CS, et al. Ambulatory care sensitive hospitalizations: elaboration of Brazilian list as a tool for measuring health system performance (Project ICSAP--Brazil). Cad Saúde Pública. 2009;25(6):1337-49.

**Table S2 – Leading primary causes of ACSC admissions**

| **Group** | **Leading five ICD primary diagnoses (number of admissions and percentage of group)** |
| --- | --- |
| All ACSCs | O23 - Infections of genitourinary tract in pregnancy (2586; 16.5%), I64 - Stroke, not specified as haemorrhage or infarction (1611; 10.3%), I50 - Heart failure (1421; 9.1%), N39 - Other disorders of urinary system (1025; 6.6%), L03 - Cellulitis (659; 4.2%) |
|  |  |
| Vaccine preventable diseases | A15 - Respiratory tuberculosis, bacteriologically and histologically confirmed (395; 69.2%), B20 - Human immunodeficiency virus [HIV] disease resulting in infectious and parasitic diseases (42; 7.4%), A18 - Tuberculosis of other organs (19; 3.3%), A19 - Miliary tuberculosis (17; 3.0%), B24 - Unspecified human immunodeficiency virus [HIV] disease (17; 3.0%) |
| Infectious gastroenteritis | E86 - Volume depletion (69; 30.4%), A09 - Other gastroenteritis and colitis of infectious and unspecified origin (46; 20.3%), A04 - Other bacterial intestinal infections (30; 13.2%), A08 - Viral and other specified intestinal infections (15; 6.6%), C53 - Malignant neoplasm of cervix uteri (7; 3.1%) |
| Anaemia | D50 - Iron deficiency anaemia (77; 90.6%), N39 - Other disorders of urinary system (2; 2.4%), B20 - Human immunodeficiency virus [HIV] disease resulting in infectious and parasitic diseases (1; 1.2%), C53 - Malignant neoplasm of cervix uteri (1; 1.2%), I05 - Rheumatic mitral valve diseases (1; 1.2%) |
| Nutritional deficiencies | E43 - Unspecified severe protein-energy malnutrition (118; 41.4%), E46 - Unspecified protein-energy malnutrition (98; 34.4%), E44 - Protein-energy malnutrition of moderate and mild degree (22; 7.7%), B20 - Human immunodeficiency virus [HIV] disease resulting in infectious and parasitic diseases (5; 1.8%), L98 - Other disorders of skin and subcutaneous tissue, not elsewhere classified (5; 1.8%) |
| Ear, nose, throat infections | J06 - Acute upper respiratory infections of multiple and unspecified sites (31; 39.2%), J03 - Acute tonsillitis (27; 34.2%), H66 - Suppurative and unspecified otitis media (6; 7.6%), J01 - Acute sinusitis (5; 6.3%), B20 - Human immunodeficiency virus [HIV] disease resulting in infectious and parasitic diseases (3; 3.8%) |
| Bacterial infections | J15 - Bacterial pneumonia, not elsewhere classified (624; 87.8%), J13 - Pneumonia due to Streptococcus pneumoniae (12; 1.7%), J18 - Pneumonia, organism unspecified (10; 1.4%), A41 - Other sepsis (9; 1.3%), B20 - Human immunodeficiency virus [HIV] disease resulting in infectious and parasitic diseases (9; 1.3%) |
| Asthma | J45 - Asthma (62; 79.5%), J18 - Pneumonia, organism unspecified (5; 6.4%), B24 - Unspecified human immunodeficiency virus [HIV] disease (2; 2.6%), J15 - Bacterial pneumonia, not elsewhere classified (2; 2.6%), J46 - Status asthmaticus (2; 2.6%) |
| Lower respiratory diseases | J44 - Other chronic obstructive pulmonary disease (254; 69.4%), J43 - Emphysema (20; 5.5%), J47 - Bronchiectasis (18; 4.9%), J18 - Pneumonia, organism unspecified (14; 3.8%), J21 - Acute bronchiolitis (13; 3.6%) |
| Hypertension | I10 - Essential (primary) hypertension (434; 57.3%), I11 - Hypertensive heart disease (73; 9.6%), I64 - Stroke, not specified as haemorrhage or infarction (59; 7.8%), I50 - Heart failure (25; 3.3%), I21 - Acute myocardial infarction (19; 2.5%) |
| Angina | I20 - Angina pectoris (309; 95.4%), I24 - Other acute ischaemic heart diseases (8; 2.5%), I21 - Acute myocardial infarction (4; 1.2%), I13 - Hypertensive heart and renal disease (1; 0.3%), I42 - Cardiomyopathy (1; 0.3%) |
| Heart failure | I50 - Heart failure (1421; 85.3%), J81 - Pulmonary oedema (207; 12.4%), A41 - Other sepsis (9; 0.5%), B57 - Chagas disease (4; 0.2%), J15 - Bacterial pneumonia, not elsewhere classified (4; 0.2%) |
| Cerebrovascular diseases | I64 - Stroke, not specified as haemorrhage or infarction (1611; 74.2%), I63 - Cerebral infarction (209; 9.6%), G45 - Transient cerebral ischaemic attacks and related syndromes (142; 6.5%), I69 - Sequelae of cerebrovascular disease (71; 3.3%), I67 - Other cerebrovascular diseases (38; 1.7%), |
| Diabetes | E14 - Unspecified diabetes mellitus (550; 40.7%), E10 - Type 1 diabetes mellitus (320; 23.7%), E11 - Type 2 diabetes mellitus (227; 16.8%), E13 - Other specified diabetes mellitus (141; 10.4%), I50 - Heart failure (13; 1.0%) |
| Epilepsy | G40 - Epilepsy (315; 91.8%), G41 - Status epilepticus (20; 5.8%), O99 - Other maternal diseases classifiable elsewhere but complicating pregnancy, childbirth and the puerperium (2; 0.6%), A31 - Infection due to other mycobacteria (1; 0.3%), B22 - Human immunodeficiency virus [HIV] disease resulting in other specified diseases (1; 0.3%) |
| Kidney and UTIs | N39 - Other disorders of urinary system (1025; 62.0%), N11 - Chronic tubulo-interstitial nephritis (256; 15.5%), N10 - Acute tubulo-interstitial nephritis (93; 5.6%), N30 - Cystitis (57; 3.5%), C53 - Malignant neoplasm of cervix uteri (53; 3.2%) |
| Skin infections | L03 - Cellulitis (659; 41.7%), A46 - Erysipelas (540; 34.1%), L02 - Cutaneous abscess, furuncle and carbuncle (221; 14.0%), L08 - Other local infections of skin and subcutaneous tissue (96; 6.1%), A49 - Bacterial infection of unspecified site (7; 0.4%) |
| Female pelvic inflammatory disease | N73 - Other female pelvic inflammatory diseases (259; 47.9%), N70 - Salpingitis and oophoritis (211; 39.0%), N75 - Diseases of Bartholin gland (46; 8.5%), N76 - Other inflammation of vagina and vulva (7; 1.3%), B24 - Unspecified human immunodeficiency virus [HIV] disease (2; 0.4%) |
| Gastrointestinal ulcer | K92 - Other diseases of digestive system (293; 55.5%), K25 - Gastric ulcer (148; 28.0%), K26 - Duodenal ulcer (46; 8.7%), K27 - Peptic ulcer, site unspecified (15; 2.8%), K28 - Gastrojejunal ulcer (8; 1.5%) |
| Diseases related to pregnancy | O23 - Infections of genitourinary tract in pregnancy (2586; 96.0%), O80 - Single spontaneous delivery (30; 1.1%), O47 - False labour (18; 0.7%), O99 - Other maternal diseases classifiable elsewhere but complicating pregnancy, childbirth and the puerperium (16; 0.6%), O62 - Abnormalities of forces of labour (8; 0.3%) |

N.B. Some only primary causes reported. Some ACSCs coded within secondary causes of admission, hence not all primary cases may reflect ACSC coding (E.g. B20 - Human immunodeficiency virus [HIV] disease resulting in infectious and parasitic diseases).

**Table S3 – Results from multilevel Poisson regression models on ACSC admissions with categories of FHS use**

|  | **ACSC Admissions** | |
| --- | --- | --- |
|  | **RR** | **95%CI** |
| FHS use |  |  |
| No consultations | 1 (Ref) |  |
| 1 consultation | 1.229*** | 1.139,1.326 |
| 2 consultations | 1.042 | 0.945,1.148 |
| 3 consultations | 0.967 | 0.851,1.098 |
| 4 or 5 consultations | 0.816** | 0.713,0.935 |
| 6-9 consultations | 0.649*** | 0.544,0.775 |
| 10-19 consultations | 0.583*** | 0.434,0.783 |
| 20+ consultations | 0.748 | 0.337,1.662 |
| Sex |  |  |
| Male | 1 (Ref) |  |
| Female | 1.191*** | 1.139,1.245 |
| Race |  |  |
| White | 1 (Ref) |  |
| Black | 1.275*** | 1.205,1.350 |
| Parda | 1.059* | 1.010,1.110 |
| Other | 1.097 | 0.950,1.267 |
| Age (years) |  |  |
| 15-19 | 1 (Ref) |  |
| 20-24 | 2.121*** | 1.904,2.361 |
| 25-29 | 2.025*** | 1.803,2.273 |
| 30-34 | 1.630*** | 1.442,1.842 |
| 35-39 | 1.547*** | 1.364,1.755 |
| 40-44 | 1.727*** | 1.519,1.963 |
| 45-49 | 2.460*** | 2.172,2.786 |
| 50-59 | 3.828*** | 3.431,4.272 |
| 60-69 | 7.111*** | 6.361,7.950 |
| 70+ | 14.419*** | 12.791,16.255 |
| Education level |  |  |
| Preschool/Literacy/None | 1 (Ref) |  |
| Elementary school | 0.901** | 0.844,0.961 |
| High school | 0.634*** | 0.587,0.685 |
| Higher education | 0.446*** | 0.335,0.595 |
| Disability |  |  |
| No | 1 (Ref) |  |
| Yes | 2.169*** | 2.021,2.327 |
| Unemployed |  |  |
| No | 1 (Ref) |  |
| Yes | 1.121*** | 1.070,1.175 |
| Income Quintiles |  |  |
| Q1 (<R$45; poorest) | 1 (Ref) |  |
| Q2 (R$$45-74) | 0.870*** | 0.816,0.928 |
| Q3 (R$75-114) | 0.825*** | 0.773,0.880 |
| Q4 (R$114-197) | 0.776*** | 0.726,0.830 |
| Q5 (R$197+; richest) | 0.716*** | 0.664,0.771 |
| Number of children in family |  |  |
| None | 1 (Ref) |  |
| One | 0.987 | 0.936,1.040 |
| Two | 1.199*** | 1.122,1.280 |
| Three of more | 1.445*** | 1.334,1.564 |
| Bolsa Familia claiming family? |  |  |
| No | 1 (Ref) |  |
| Yes | 1.257*** | 1.192,1.325 |
| Per capita medicine expenditure |  |  |
| None | 1 (Ref) |  |
| 0-R$50 | 1.081** | 1.021,1.146 |
| >R$50 | 1.196*** | 1.118,1.280 |
| Formal employment in family |  |  |
| No | 1 (Ref) |  |
| Yes | 0.910** | 0.860,0.963 |
|  |  |  |
| Total individuals | 1240009 |  |

Results from adjusted multilevel Poisson regression models. Models additionally adjusted for time effects and include a person-year offset. * p<0.05; ** p<0.01; *** p<0.001

**Table S4 – Results from multilevel regression Poisson regression models on admissions and readmissions excluding 90-days before and after first FHS use**

|  | **ACSC Admissions** | | **30-day readmission (any nonbirth cause)** | | **30-day readmission (ACSC only)** | |
| --- | --- | --- | --- | --- | --- | --- |
|  | **RR** | **RR** | **95%CI** | **RR** | **95%CI** | **RR** |
| FHS use | 0.962*** | 0.947,0.978 | 0.352*** | 0.309,0.401 | 0.396*** | 0.295,0.533 |
| Sex |  |  |  |  |  |  |
| Male | 1 (Ref) |  | 1 (Ref) |  |  |  |
| Female | 1.187*** | 1.134,1.243 | 0.698*** | 0.570,0.856 | 1.070 | 0.825,1.388 |
| Race |  |  |  |  |  |  |
| White | 1 (Ref) |  | 1 (Ref) |  |  |  |
| Black | 1.282*** | 1.209,1.360 | 1.490** | 1.161,1.913 | 1.398* | 1.020,1.916 |
| Parda | 1.063* | 1.013,1.117 | 1.073 | 0.846,1.361 | 0.787 | 0.593,1.043 |
| Other | 1.114 | 0.961,1.291 | 1.296 | 0.749,2.242 | 1.116 | 0.521,2.390 |
| Age (years) |  |  |  |  |  |  |
| 15-19 | 1 (Ref) |  | 1 (Ref) |  |  |  |
| 20-24 | 2.263*** | 2.017,2.539 | 2.484* | 1.129,5.465 | 1.434 | 0.747,2.753 |
| 25-29 | 2.205*** | 1.949,2.495 | 1.614 | 0.426,6.123 | 1.007 | 0.478,2.121 |
| 30-34 | 1.803*** | 1.585,2.052 | 2.985* | 1.294,6.887 | 1.01 | 0.488,2.091 |
| 35-39 | 1.691*** | 1.480,1.931 | 5.267*** | 2.298,12.069 | 0.824 | 0.392,1.732 |
| 40-44 | 1.918*** | 1.675,2.196 | 7.122*** | 3.039,16.690 | 0.977 | 0.436,2.188 |
| 45-49 | 2.768*** | 2.427,3.155 | 15.078*** | 6.577,34.564 | 2.002 | 0.962,4.170 |
| 50-59 | 4.339*** | 3.862,4.874 | 25.007*** | 11.896,52.567 | 3.621*** | 1.815,7.226 |
| 60-69 | 8.239*** | 7.324,9.269 | 68.445*** | 33.108,141.501 | 10.044*** | 4.998,20.184 |
| 70+ | 16.875*** | 14.881,19.137 | 101.248*** | 47.543,215.619 | 15.865*** | 7.583,33.193 |
| Education level |  |  |  |  |  |  |
| Preschool/Literacy/None | 1 (Ref) |  | 1 (Ref) |  |  |  |
| Elementary school | 0.894** | 0.837,0.956 | 1.003 | 0.750,1.342 | 0.882 | 0.597,1.302 |
| High school | 0.630*** | 0.582,0.682 | 0.543** | 0.373,0.791 | 0.495** | 0.306,0.799 |
| Higher education | 0.461*** | 0.341,0.622 | 0.480 | 0.168,1.372 | 0.079* | 0.011,0.581 |
| Disability |  |  |  |  |  |  |
| No | 1 (Ref) |  | 1 (Ref) |  |  |  |
| Yes | 2.197*** | 2.043,2.362 | 2.406*** | 1.884,3.073 | 1.973*** | 1.355,2.874 |
| Unemployed |  |  |  |  |  |  |
| No | 1 (Ref) |  | 1 (Ref) |  |  |  |
| Yes | 1.133*** | 1.080,1.189 | 1.935*** | 1.550,2.415 | 1.916*** | 1.447,2.536 |
| Income Quintiles |  |  |  |  |  |  |
| Q1 (<R$45; poorest) | 1 (Ref) |  | 1 (Ref) |  |  |  |
| Q2 (R$$45-74) | 0.864*** | 0.808,0.923 | 1.212 | 0.872,1.683 | 0.817 | 0.541,1.234 |
| Q3 (R$75-114) | 0.825*** | 0.772,0.883 | 0.813 | 0.569,1.161 | 0.939 | 0.626,1.407 |
| Q4 (R$114-197) | 0.764*** | 0.713,0.819 | 1.049 | 0.706,1.558 | 0.766 | 0.498,1.178 |
| Q5 (R$197+; richest) | 0.693*** | 0.642,0.749 | 0.957 | 0.689,1.328 | 0.853 | 0.564,1.291 |
| Number of children in family |  |  |  |  |  |  |
| None | 1 (Ref) |  | 1 (Ref) |  |  |  |
| One | 0.971 | 0.919,1.026 | 1.082 | 0.825,1.420 | 1.266 | 0.922,1.738 |
| Two | 1.184*** | 1.106,1.268 | 1.141 | 0.806,1.617 | 1.358 | 0.911,2.022 |
| Three of more | 1.439*** | 1.325,1.563 | 1.380 | 0.864,2.204 | 1.336 | 0.798,2.239 |
| Bolsa Familia claiming family? |  |  |  |  |  |  |
| No | 1 (Ref) |  | 1 (Ref) |  |  |  |
| Yes | 1.261*** | 1.194,1.332 | 1.279 | 0.983,1.665 | 1.318 | 0.974,1.784 |
| Per capita medicine expenditure |  |  |  |  |  |  |
| None | 1 (Ref) |  | 1 (Ref) |  |  |  |
| 0-R$50 | 1.074* | 1.011,1.141 | 0.838 | 0.644,1.092 | 0.847 | 0.600,1.196 |
| >R$50 | 1.206*** | 1.126,1.293 | 1.054 | 0.699,1.591 | 0.783 | 0.517,1.186 |
| Formal employment in family |  |  |  |  |  |  |
| No | 1 (Ref) |  | 1 (Ref) |  |  |  |
| Yes | 0.898*** | 0.846,0.953 | 0.948 | 0.724,1.241 | 0.778 | 0.543,1.116 |
|  |  |  |  |  |  |  |
| Total individuals | 1239760 |  | 1239760 |  | 1239760 |  |

Results from adjusted multilevel Poisson regression models. Models additionally adjusted for time effects and include a person-year offset. * p<0.05; ** p<0.01; *** p<0.001

**Table S5 – Results from multilevel regression Poisson regression models on elective admissions from ACSCs**

|  | **ACSC Admissions** | |
| --- | --- | --- |
|  | **RR** | **RR** |
| FHS use | 0.962** | 0.935,0.989 |
| Sex |  |  |
| Male | 1 (Ref) |  |
| Female | 0.978 | 0.901,1.062 |
| Race |  |  |
| White | 1 (Ref) |  |
| Black | 1.144* | 1.029,1.272 |
| Parda | 0.929 | 0.849,1.015 |
| Other | 1.054 | 0.824,1.347 |
| Age (years) |  |  |
| 15-19 | 1 (Ref) |  |
| 20-24 | 2.077*** | 1.611,2.678 |
| 25-29 | 2.345*** | 1.781,3.088 |
| 30-34 | 2.538*** | 1.919,3.357 |
| 35-39 | 2.740*** | 2.095,3.585 |
| 40-44 | 3.317*** | 2.545,4.324 |
| 45-49 | 4.788*** | 3.688,6.217 |
| 50-59 | 7.224*** | 5.695,9.163 |
| 60-69 | 12.320*** | 9.665,15.703 |
| 70+ | 16.651*** | 12.852,21.571 |
| Education level |  |  |
| Preschool/Literacy/None | 1 (Ref) |  |
| Elementary school | 0.907 | 0.802,1.025 |
| High school | 0.665*** | 0.574,0.771 |
| Higher education | 0.813 | 0.534,1.240 |
| Disability |  |  |
| No | 1 (Ref) |  |
| Yes | 1.975*** | 1.749,2.229 |
| Unemployed |  |  |
| No | 1 (Ref) |  |
| Yes | 1.168*** | 1.065,1.280 |
| Income Quintiles |  |  |
| Q1 (<R$45; poorest) | 1 (Ref) |  |
| Q2 (R$$45-74) | 1.026 | 0.899,1.171 |
| Q3 (R$75-114) | 0.858* | 0.749,0.983 |
| Q4 (R$114-197) | 0.961 | 0.842,1.096 |
| Q5 (R$197+; richest) | 0.793** | 0.688,0.913 |
| Number of children in family |  |  |
| None | 1 (Ref) |  |
| One | 0.883* | 0.795,0.981 |
| Two | 0.973 | 0.850,1.115 |
| Three of more | 1.281** | 1.081,1.518 |
| Bolsa Familia claiming family? |  |  |
| No | 1 (Ref) |  |
| Yes | 1.054 | 0.955,1.162 |
| Per capita medicine expenditure |  |  |
| None | 1 (Ref) |  |
| 0-R$50 | 1.283*** | 1.154,1.427 |
| >R$50 | 1.603*** | 1.435,1.792 |
| Formal employment in family |  |  |
| No | 1 (Ref) |  |
| Yes | 0.791*** | 0.708,0.885 |
|  |  |  |
| Total individuals | 1240009 |  |

Results from adjusted multilevel Poisson regression models. Models additionally adjusted for time effects and include a person-year offset. * p<0.05; ** p<0.01; *** p<0.001

**Table S6 – Results from multilevel regression Poisson regression models on admissions and readmissions without IPTW**

|  | **ACSC Admissions** | | **30-day readmission (any nonbirth cause)** | | **30-day readmission (ACSC only)** | |
| --- | --- | --- | --- | --- | --- | --- |
|  | **RR** | **RR** | **95%CI** | **RR** | **95%CI** | **RR** |
| FHS use | 0.944*** | 0.931,0.958 | 0.370*** | 0.278,0.494 | 0.331*** | 0.262,0.419 |
| Sex |  |  |  |  |  |  |
| Male | 1 (Ref) |  | 1 (Ref) |  | 1 (Ref) |  |
| Female | 1.193*** | 1.146,1.242 | 1.108 | 0.881,1.394 | 0.767** | 0.648,0.907 |
| Race |  |  |  |  |  |  |
| White | 1 (Ref) |  | 1 (Ref) |  | 1 (Ref) |  |
| Black | 1.290*** | 1.224,1.360 | 1.556** | 1.175,2.061 | 1.521*** | 1.219,1.899 |
| Parda | 1.068** | 1.022,1.116 | 0.793 | 0.614,1.024 | 1.017 | 0.839,1.233 |
| Other | 1.057 | 0.929,1.204 | 1.116 | 0.552,2.257 | 1.255 | 0.733,2.150 |
| Age (years) |  |  |  |  |  |  |
| 15-19 | 1 (Ref) |  | 1 (Ref) |  | 1 (Ref) |  |
| 20-24 | 2.197*** | 1.990,2.427 | 1.525 | 0.873,2.661 | 2.394** | 1.237,4.635 |
| 25-29 | 2.096*** | 1.886,2.330 | 1.075 | 0.572,2.023 | 1.791 | 0.862,3.721 |
| 30-34 | 1.756*** | 1.567,1.967 | 1.186 | 0.626,2.248 | 3.607*** | 1.743,7.467 |
| 35-39 | 1.673*** | 1.491,1.878 | 0.921 | 0.473,1.794 | 5.775*** | 2.798,11.920 |
| 40-44 | 1.834*** | 1.632,2.062 | 0.838 | 0.421,1.669 | 7.257*** | 3.470,15.176 |
| 45-49 | 2.699*** | 2.409,3.025 | 2.518** | 1.340,4.730 | 17.080*** | 8.272,35.265 |
| 50-59 | 4.477*** | 4.047,4.952 | 4.042*** | 2.214,7.377 | 26.354*** | 13.553,51.246 |
| 60-69 | 8.514*** | 7.682,9.436 | 12.395*** | 6.896,22.277 | 76.416*** | 40.763,143.253 |
| 70+ | 18.925*** | 16.947,21.133 | 18.712*** | 10.136,34.545 | 115.394*** | 60.328,220.723 |
| Education level |  |  |  |  |  |  |
| Preschool/Literacy/None | 1 (Ref) |  | 1 (Ref) |  | 1 (Ref) |  |
| Elementary school | 0.895*** | 0.843,0.950 | 0.995 | 0.711,1.393 | 1.074 | 0.841,1.371 |
| High school | 0.637*** | 0.593,0.683 | 0.544** | 0.356,0.832 | 0.592** | 0.426,0.825 |
| Higher education | 0.376*** | 0.301,0.470 | 0.074* | 0.010,0.536 | 0.332* | 0.123,0.898 |
| Disability |  |  |  |  |  |  |
| No | 1 (Ref) |  | 1 (Ref) |  | 1 (Ref) |  |
| Yes | 2.207*** | 2.063,2.362 | 2.027*** | 1.413,2.909 | 2.322*** | 1.847,2.919 |
| Unemployed |  |  |  |  |  |  |
| No | 1 (Ref) |  | 1 (Ref) |  | 1 (Ref) |  |
| Yes | 1.136*** | 1.087,1.187 | 1.925*** | 1.486,2.495 | 1.945*** | 1.600,2.365 |
| Income Quintiles |  |  |  |  |  |  |
| Q1 (<R$45; poorest) | 1 (Ref) |  | 1 (Ref) |  | 1 (Ref) |  |
| Q2 (R$$45-74) | 0.874*** | 0.824,0.928 | 0.888 | 0.612,1.288 | 1.167 | 0.870,1.566 |
| Q3 (R$75-114) | 0.831*** | 0.783,0.883 | 0.984 | 0.683,1.417 | 0.8 | 0.582,1.101 |
| Q4 (R$114-197) | 0.783*** | 0.736,0.833 | 0.782 | 0.534,1.145 | 1.035 | 0.770,1.393 |
| Q5 (R$197+; richest) | 0.706*** | 0.659,0.755 | 1.013 | 0.697,1.472 | 0.955 | 0.710,1.284 |
| Number of children in family |  |  |  |  |  |  |
| None | 1 (Ref) |  | 1 (Ref) |  | 1 (Ref) |  |
| One | 0.991 | 0.944,1.041 | 1.158 | 0.869,1.543 | 1.088 | 0.867,1.366 |
| Two | 1.213*** | 1.140,1.290 | 1.475* | 1.028,2.116 | 1.167 | 0.855,1.591 |
| Three of more | 1.493*** | 1.384,1.610 | 1.213 | 0.739,1.991 | 1.264 | 0.827,1.931 |
| Bolsa Familia claiming family? |  |  |  |  |  |  |
| No | 1 (Ref) |  | 1 (Ref) |  | 1 (Ref) |  |
| Yes | 1.320*** | 1.258,1.384 | 1.362* | 1.053,1.762 | 1.328** | 1.083,1.628 |
| Per capita medicine expenditure |  |  |  |  |  |  |
| None | 1 (Ref) |  | 1 (Ref) |  | 1 (Ref) |  |
| 0-R$50 | 1.068* | 1.011,1.128 | 0.907 | 0.663,1.242 | 0.93 | 0.730,1.186 |
| >R$50 | 1.170*** | 1.098,1.247 | 0.783 | 0.535,1.146 | 1.049 | 0.822,1.337 |
| Formal employment in family |  |  |  |  |  |  |
| No | 1 (Ref) |  | 1 (Ref) |  | 1 (Ref) |  |
| Yes | 0.929** | 0.881,0.979 | 0.841 | 0.610,1.158 | 0.989 | 0.780,1.253 |
|  |  |  |  |  |  |  |
| Total individuals | 1240009 |  | 1240009 |  | 1240009 |  |

Results from adjusted multilevel Poisson regression models. Models additionally adjusted for time effects and include a person-year offset. * p<0.05; ** p<0.01; *** p<0.001

**Table S7 – Results from multilevel regression Poisson regression models on admissions and readmissions excluding FHS consultations for healthy pregnancy**

|  | **ACSC Admissions** | | **30-day readmission (any nonbirth cause)** | | **30-day readmission (ACSC only)** | |
| --- | --- | --- | --- | --- | --- | --- |
|  | **RR** | **RR** | **95%CI** | **RR** | **95%CI** | **RR** |
| FHS use | 0.958*** | 0.942,0.973 | 0.361*** | 0.257,0.507 | 0.360*** | 0.287,0.452 |
| Sex |  |  |  |  |  |  |
| Male | 1 (Ref) |  | 1 (Ref) |  | 1 (Ref) |  |
| Female | 1.192*** | 1.140,1.246 | 1.091 | 0.848,1.403 | 0.719*** | 0.592,0.874 |
| Race |  |  |  |  |  |  |
| White | 1 (Ref) |  | 1 (Ref) |  | 1 (Ref) |  |
| Black | 1.276*** | 1.205,1.350 | 1.481* | 1.096,2.001 | 1.483** | 1.144,1.923 |
| Parda | 1.059* | 1.011,1.111 | 0.811 | 0.618,1.066 | 1.028 | 0.806,1.313 |
| Other | 1.097 | 0.950,1.268 | 1.055 | 0.497,2.241 | 1.189 | 0.689,2.053 |
| Age (years) |  |  |  |  |  |  |
| 15-19 | 1 (Ref) |  | 1 (Ref) |  | 1 (Ref) |  |
| 20-24 | 2.131*** | 1.914,2.372 | 1.331 | 0.732,2.417 | 2.139 | 0.511,8.954 |
| 25-29 | 2.036*** | 1.813,2.285 | 0.963 | 0.482,1.925 | 1.512 | 0.345,6.621 |
| 30-34 | 1.639*** | 1.450,1.852 | 1.053 | 0.534,2.078 | 3.145 | 0.686,14.420 |
| 35-39 | 1.556*** | 1.372,1.765 | 0.708 | 0.351,1.427 | 4.850* | 1.092,21.540 |
| 40-44 | 1.738*** | 1.529,1.976 | 0.836 | 0.389,1.798 | 6.774* | 1.575,29.141 |
| 45-49 | 2.480*** | 2.190,2.807 | 1.859 | 0.939,3.680 | 15.083*** | 3.582,63.515 |
| 50-59 | 3.862*** | 3.463,4.308 | 3.058*** | 1.618,5.778 | 23.431*** | 5.920,92.743 |
| 60-69 | 7.180*** | 6.425,8.024 | 9.403*** | 4.977,17.764 | 65.448*** | 16.840,254.357 |
| 70+ | 14.595*** | 12.954,16.444 | 14.700*** | 7.491,28.848 | 98.273*** | 25.280,382.025 |
| Education level |  |  |  |  |  |  |
| Preschool/Literacy/None | 1 (Ref) |  | 1 (Ref) |  | 1 (Ref) |  |
| Elementary school | 0.900** | 0.844,0.961 | 0.876 | 0.603,1.271 | 1.011 | 0.770,1.328 |
| High school | 0.634*** | 0.587,0.684 | 0.467** | 0.295,0.739 | 0.524** | 0.339,0.808 |
| Higher education | 0.445*** | 0.333,0.594 | 0.074* | 0.010,0.547 | 0.451 | 0.159,1.275 |
| Disability |  |  |  |  |  |  |
| No | 1 (Ref) |  | 1 (Ref) |  | 1 (Ref) |  |
| Yes | 2.165*** | 2.018,2.323 | 1.802** | 1.242,2.614 | 2.260*** | 1.781,2.866 |
| Unemployed |  |  |  |  |  |  |
| No | 1 (Ref) |  | 1 (Ref) |  | 1 (Ref) |  |
| Yes | 1.121*** | 1.070,1.174 | 1.947*** | 1.488,2.548 | 1.934*** | 1.577,2.373 |
| Income Quintiles |  |  |  |  |  |  |
| Q1 (<R$45; poorest) | 1 (Ref) |  | 1 (Ref) |  | 1 (Ref) |  |
| Q2 (R$$45-74) | 0.870*** | 0.816,0.928 | 0.907 | 0.610,1.351 | 1.256 | 0.878,1.796 |
| Q3 (R$75-114) | 0.825*** | 0.773,0.880 | 0.987 | 0.668,1.458 | 0.837 | 0.594,1.179 |
| Q4 (R$114-197) | 0.776*** | 0.726,0.830 | 0.801 | 0.527,1.218 | 1.066 | 0.777,1.463 |
| Q5 (R$197+; richest) | 0.716*** | 0.664,0.772 | 0.895 | 0.599,1.337 | 0.986 | 0.723,1.343 |
| Number of children in family |  |  |  |  |  |  |
| None | 1 (Ref) |  | 1 (Ref) |  | 1 (Ref) |  |
| One | 0.987 | 0.936,1.041 | 1.228 | 0.902,1.672 | 1.104 | 0.839,1.454 |
| Two | 1.199*** | 1.123,1.280 | 1.402 | 0.960,2.049 | 1.115 | 0.807,1.541 |
| Three of more | 1.445*** | 1.335,1.565 | 1.253 | 0.753,2.085 | 1.268 | 0.816,1.970 |
| Bolsa Familia claiming family? |  |  |  |  |  |  |
| No | 1 (Ref) |  | 1 (Ref) |  | 1 (Ref) |  |
| Yes | 1.257*** | 1.192,1.326 | 1.302 | 0.973,1.743 | 1.324* | 1.062,1.650 |
| Per capita medicine expenditure |  |  |  |  |  |  |
| None | 1 (Ref) |  | 1 (Ref) |  | 1 (Ref) |  |
| 0-R$50 | 1.082** | 1.021,1.146 | 0.842 | 0.604,1.174 | 0.868 | 0.673,1.118 |
| >R$50 | 1.195*** | 1.116,1.278 | 0.715 | 0.475,1.077 | 1.003 | 0.781,1.289 |
| Formal employment in family |  |  |  |  |  |  |
| No | 1 (Ref) |  | 1 (Ref) |  | 1 (Ref) |  |
| Yes | 0.910** | 0.859,0.963 | 0.827 | 0.587,1.163 | 0.984 | 0.760,1.275 |
|  |  |  |  |  |  |  |
| Total individuals | 1240009 |  | 1240009 |  | 1240009 |  |

Results from adjusted multilevel Poisson regression models. Models additionally adjusted for time effects and include a person-year offset. * p<0.05; ** p<0.01; *** p<0.001

Excludes FHS consultations relating to healthy, normal pregnancy (ICD10 codes Z32-Z34, Z36; and International Classification of Primary Care (ICPC-2) produced codes W01 and W78).

**Table S8 – Deaths in Rio de Janeiro by location of death (counts and proportions) 2010-2017**

| **Deaths:** |  | |  | |  | |  | |  | |  | |  | |  |
| --- | --- | --- | --- | --- | --- | --- | --- | --- | --- | --- | --- | --- | --- | --- | --- |
|  | 2010 | 2011 | | 2012 | | 2013 | | 2014 | | 2015 | | 2016 | | 2017 | |
| Hospital/health facility | 43776 | 42532 | | 42658 | | 43251 | | 44108 | | 44928 | | 47229 | | 44738 | |
| Home | 7563 | 7387 | | 7149 | | 7730 | | 7544 | | 7828 | | 8343 | | 8203 | |
| Public area | 877 | 516 | | 442 | | 394 | | 389 | | 341 | | 335 | | 317 | |
| Other/missing | 1630 | 2089 | | 2014 | | 1978 | | 1955 | | 1949 | | 2131 | | 2124 | |
|  |  |  | |  | |  | |  | |  | |  | |  | |
| **As percentage of total deaths:** |  |  | |  | |  | |  | |  | |  | |  | |
|  | 2010 | 2011 | | 2012 | | 2013 | | 2014 | | 2015 | | 2016 | | 2017 | |
| Hospital/health facility | 81.3% | 81.0% | | 81.6% | | 81.1% | | 81.7% | | 81.6% | | 81.4% | | 80.8% | |
| Home | 14.0% | 14.1% | | 13.7% | | 14.5% | | 14.0% | | 14.2% | | 14.4% | | 14.8% | |
| Public area | 1.6% | 1.0% | | 0.8% | | 0.7% | | 0.7% | | 0.6% | | 0.6% | | 0.6% | |
| Other/missing | 3.0% | 4.0% | | 3.9% | | 3.7% | | 3.6% | | 3.5% | | 3.7% | | 3.8% | |

Source: DATASUS/TABNET <https://datasus.saude.gov.br/informacoes-de-saude-tabnet/> . Death obtained for the municipality of Rio de Janeiro by location of death as recorded on the death certificate.

**Table S9 – Hospitals and hospital beds in Rio de Janeiro (2010-2017)**

|  | 2010 | 2011 | 2012 | 2013 | 2014 | 2015 | 2016 | 2017 |
| --- | --- | --- | --- | --- | --- | --- | --- | --- |
| **Hospitals:** |  |  |  |  |  |  |  |  |
| Public (SUS) | 94 | 92 | 102 | 100 | 97 | 94 | 94 | 91 |
| Private | 188 | 188 | 183 | 175 | 182 | 144 | 123 | 138 |
|  |  |  |  |  |  |  |  |  |
| **Hospital beds:** |  |  |  |  |  |  |  |  |
| Public (SUS) | 12831 | 12498 | 12633 | 11523 | 11230 | 10492 | 9858 | 9351 |
| Private | 10184 | 11173 | 11274 | 11339 | 11088 | 7925 | 7087 | 7343 |

Source: DATASUS/TABNET <https://datasus.saude.gov.br/informacoes-de-saude-tabnet/> . Hospitals include all health establishments that provide hospitalisations/admissions. Data for the municipality (city) of Rio de Janeiro.
